# Optimizing the Process of Spot Welding of Polycarbonate-Matrix-Based Unidirectional (UD) Thermoplastic Composite Tapes

**DOI:** 10.3390/polym15092182

**Published:** 2023-05-04

**Authors:** Janos Birtha, Christian Marschik, Eva Kobler, Klaus Straka, Georg Steinbichler, Sven Schlecht, Paul Zwicklhuber

**Affiliations:** 1 Institute of Polymer Injection Moulding and Process Automation, Johannes Kepler University Linz, Altenbergerstraße 69, 4040 Linz, Austria; 2 Competence Center CHASE GmbH, Altenbergerstraße 69, 4040 Linz, Austria; 3 Covestro Deutschland AG, Kaiser-Wilhelm-Allee 60, 51373 Leverkusen, Germany; 4 ENGEL AUSTRIA GmbH, Ludwig-Engel-Straße 1, 4311 Schwertberg, Austria

**Keywords:** thermoplastic composites, unidirectional tapes, spot-welding, optimization

## Abstract

The aim of this work was to optimize spot welding of unidirectional tapes made of polycarbonate and carbon fibers. Three studies were performed to investigate the influences of various welding conditions on the quality of the welded spot. First, we used a full factorial experimental design to analyze the influence of temperature and time on the welds’ tensile stress at break. Second, we repeated the experiment with optimized settings and conditions. Finally, we adopted a central composite design (CCD) to investigate the stability of the process. Our results show that temperature had the greatest influence on weld quality. The maximum tensile stress achieved was 23 MPa. Using a relatively high temperature for a short welding time resulted in self-cleaning of the welding head and in a faster and more stable process, and gel permeation chromatography (GPC) confirmed that these conditions caused no additional degradation.

## 1. Introduction

The goal of lightweight design—to achieve adequate mechanical performance at minimal weight—can be realized with thermoplastic composites. The increased need for lightweight design is reflected in the global market growth of composites, as from 2015 to 2020 the demand increased by 25% [1]. It has been estimated that 50% of the market share of these materials is accounted for by the automotive industry [2]. Continuous-fiber-reinforced thermoplastic composites have numerous advantages over their thermoset counterparts. Since they do not require curing, thermoplastics can be remelted multiple times [3,4,5,6]. Further, they exhibit improved damage resistance [7], infinite shelf life [7,8], and excellent vibration-dampening ability [3]. Using compression and injection molding for production allows greater freedom of design and functionalization at low production costs [8,9].

**Figure 1:**
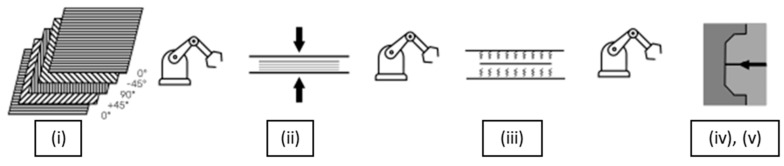
Schematic representation of the thermoplastic production cell: (**i**) tape laying, (**ii**) consolidation, (**iii**) preheating, (**iv**) forming and, optionally, (**v**) overmolding [10].

A novel method for producing thermoplastic composites is the so-called *thermoplastic composite production cell*, which enables fully automated manufacturing of thermoplastic composite parts from UD tapes. It consists of five process steps: (i) tape laying, (ii) consolidation, (iii) preheating, (iv) forming and, optionally, (v) overmolding (Figure 1).

**Figure 2:**
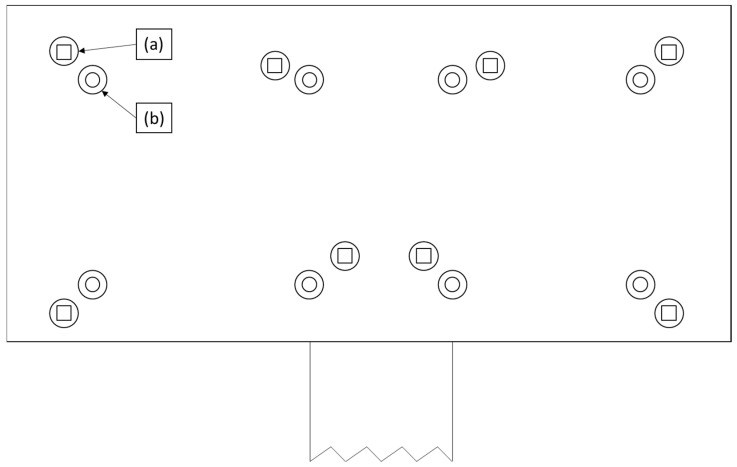
The end-of-arm tool of an industrial robot, equipped with (**a**) welding units and (**b**) suction cups.

In the first step, UD tapes of specified dimensions are laid down and spot-welded in layers in a predefined position and orientation by two industrial robots. The end-of-arm tools attached to the robots are equipped with numerous welding (Figure 2a) and suction (Figure 2b) units. The semi-finished product is then moved to the consolidation stage, where the tape stack is first compressed by a heating press. At this stage, the tapes are melted to form a bond between the individual layers. The part is then moved to a cooling press, where consolidation is completed. The consolidated tape stack is subsequently transported to an infrared oven, which preheats the material above the glass-transition or melting temperature of the matrix material. In the next step, the preheated part is placed in the mold of an injection machine, which upon closing forms the preheated plate into the desired shape; depending on the mold design, ribs and bosses can be injected onto the formed plate. After cooling, the finished part is retrieved.

The main aim of this work is to optimize the spot-welding process, specifically the first step of the production cell, using a lab-scale experimental set-up. The robot heads which place the UD tapes on top of each other are equipped with multiple 4 × 4 mm^2^ spot welders. The heat and pressure exerted by these welding units bonds the tapes locally to form a tape stack. The quality of this bonding is influenced by temperature, pressure, and time [11]. To increase product quality and reduce cycle time, these process parameters must be optimized within boundary conditions, such as those imposed by the characteristics of the matrix material.

The optimal process window for the welding operation has to be defined considering the following points. First, the welded spots must be strong enough to ensure that the stack remains intact during transportation. This requires reaching a sufficiently high welding temperature for a sufficiently long time. Second, material degradation at the welded spot must be avoided, as it may decrease the mechanical performance of the final component [12]. This sets the upper limits of the temperature and time ranges of the welding process. Third, a reduction in welding time increases the productivity of the tape-laying cell. A relatively low temperature with long bonding time would be an option, but at the industrial scale a faster process with higher temperature is preferred because more tape stacks can be produced within the same amount of time. Lastly, fiber damage and changes in fiber orientation must be avoided during the welding process.

## 2. Literature Review: Techniques for Joining Thermoplastic Composites

Thermoplastic composite joining techniques can be classified into three broad groups: (i) adhesive bonding, (ii) mechanical fastening, and (iii) fusion bonding [13,14].

Adhesive bonds require application of a glue between two surfaces, which diffuses into the substrate and forms a bond based on electrostatic and van der Waals forces [15]. The adhesives used for this purpose are usually thermosets, such as epoxy, acrylic, and formaldehyde resin-based materials [16].

According to Banae et al. [17], the quality of the adhesive bonds is influenced by surface preparation, the joint configuration, the properties of the glue, and environmental factors. Although adhesive bonds provide many advantages, there are also certain limitations [15]. Due to their low surface energy, polarity, and reactivity, thermoplastic materials cannot easily be joined with thermosets [15]. To increase bond quality, additional surface treatment is required, such as corona discharge, plasma treatment, and acid etches [18]. Further, bonding of multiple tapes is not effective, as thickness variations produced by the glues have a profound impact on consolidation quality at a later stage. Adhesive bonding also restricts the areas for tape consolidation.

Mechanical fastening involves riveting, clamping, and bolting two structures together [14]. Examples of mechanically fastened parts can be found in aircraft components, where joining a thermoplastic with a thermoset composite is economically preferable [19]. However, stress concentrations in combination with delamination caused by drilling and the difference in thermal expansion coefficients between the parts used for fastening and the substrates are a few problems that arise using this technique [14,20].

The main characteristic of fusion bonding is that energy from an external source is used to create a bond between two surfaces. The external energy increases the mobility of the molecular chains, which in turn decreases the viscosity of the material. When pressure is applied to the heated parts, the macromolecules form an entangled network, thus creating a bond between the two surfaces [21]. Depending on the source of energy, there are several fusion bonding techniques available.

The usefulness of fusion bonding depends on the geometry and composition of the components to be joined. For example, microwave welding allows whole components—in theory of any shape—to be welded [22]. However, depending on the application, creating the bond may require a high level of energy [23]. Laser transmission welding, in contrast, offers the advantage that laser radiation can pass through semi-transparent materials, thus enabling welding in hard-to-reach areas. The application field of this technique, however, is limited by the optical properties of the semi-transparent material. Depending on the matrix material and the amount of glass fiber content, radiation may scatter, which decreases the rate of energy delivered to the interface [24,25].

A fast method for achieving fusion bonds is ultrasonic, which requires no additional substances. This technique uses the friction between two surfaces as they slide against each other to generate heat [26]. However, the maximum thickness of parts is limited to 3 mm, and the mechanical properties of the matrix material may have an adverse effect on the quality of the weld, for instance, in terms of stiffness and the dampening property of the polymer [13]. Spot welding, which is the technique analyzed in this paper, uses a heated plate to provide heat and pressure to the interface between two parts. In contrast to other methods, it is a simple and economically favorable way of joining thermoplastic composites; however, the risk of surface contamination is disadvantageous [26]. As Yousepour et al. have pointed out, contamination may also be caused by the molten matrix material sticking to the hot plate [14].

Most optimization studies that investigated welding of thermoplastic composites evaluated the performance of the welded stack by mechanical testing, most commonly by lap shear tests. Ahmadi et al. used the ASTM D5868 (10 October 2002) standard to prepare samples for tensile shear testing. They optimized the friction stir welding process in terms of rotational speed, welding speed, and tilt angle [27]. Similarly, Bhudolia et al. conducted lap shear tests based on the same standard. They also evaluated the process settings that produced the highest lap shear strength [28]. Zhang et al. optimized their process based on lap shear tests performed according to the ASTM 1002 standard. In addition, they examined the failure modes of the fractured bond [29]. Huang et al. reviewed the potential of joining thermoplastic polymers and polymer composites together by means of friction stir welding based on shear strength. They concluded that low thermal conductivity, crystallinity, and voids are the main factors that have a negative impact on mechanical properties [30]. These may also influence the quality of the weld when spot welders are used; however, investigating these influences on spot welding was outside the scope of the present study. The ASTM 1002 standard was also employed in two other studies, which analyzed the optimal settings of resistance welders [31,32]. Dubé et al. determined the apparent lap shear strength based on the ASTM 3528 standard, performed fatigue tests, and also observed the various failure modes [33]. A number of studies have applied similar mechanical testing to increase our understanding of various processes for welding thermoplastic composites [34,35,36,37,38].

While the primary target variable in previous optimization studies has been the mechanical performance of the welded spot, the influences of temperature and time on degradation have received less attention. Colak et al. defined their process window for resistance welding based on the degree of bonding (DoB) and weight loss. The former describes the bond strength that develops at the interface of two surfaces, while the latter relates to degradation of the matrix material. They determined the maximum weight loss allowed to be 0.01% of the joined components, which served as an upper boundary for optimization in their welding study [39]. Talbot et al. set the maximum welding temperature to 450 °C, the degradation temperature of polyether ether ketone (PEEK). They also found that the clamping distance (distance between the edge of the weld and the electrical connector of a resistance welding set-up) influences local overheating of the weld’s edges and thus local degradation of the material [31]. Shi et al. specified the boundary of their process window such that the degree of degradation would be smaller than 0.1% [32]. Villegas et al. investigated how thermal degradation can be avoided when thermoplastic composites are welded to thermoset composites. Based on Fourier transform infrared (FTIR) spectral analysis, they concluded that heating time must be very short (less than 1 s) to prevent degradation of the thermoset material [19].

In this work, three studies were conducted to evaluate the welding of UD tapes made of polycarbonate and carbon fibers (PC/CF). The welding technique used is similar to the hot-plate method, but rather than putting the heated plate between the surfaces, heat and pressure were applied to the top of two UD tapes. We started with a screening test that involved a design of experiments (DoE) with ranges of temperatures and welding times, the results and observations from which were then applied to a second series of experiments, in which the optimal process settings for spot welding were investigated. Finally, we studied the effects of relatively high temperatures and short welding times. To optimize the process, the influences of temperature and time on mechanical performance and degradation were investigated.

## 3. Experimental Procedures

### 3.1. Material

We used PC/CF UD tapes with a fiber volume content of roughly 44% in our experiments. Table 1 provides an overview of the properties of the matrix and fiber materials.

According to the manufacturer, the glass transition temperature of the PC matrix material was 145 °C, which served as the lower boundary for the processing window. The tapes were cut to be 100 mm long and 50 mm wide.

### 3.2. Experimental Set-Up

**Figure 3:**
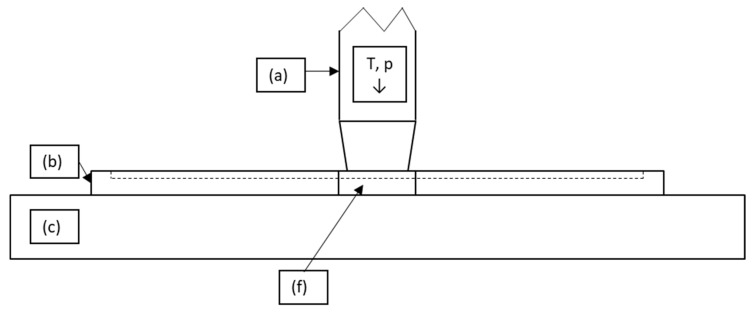
Schematic representation of the lab-scale experimental set-up seen from the front: (**a**) welding unit, (**b**) cavity, (**c**) welding table, (**f**) porous metal piece.

**Figure 4:**
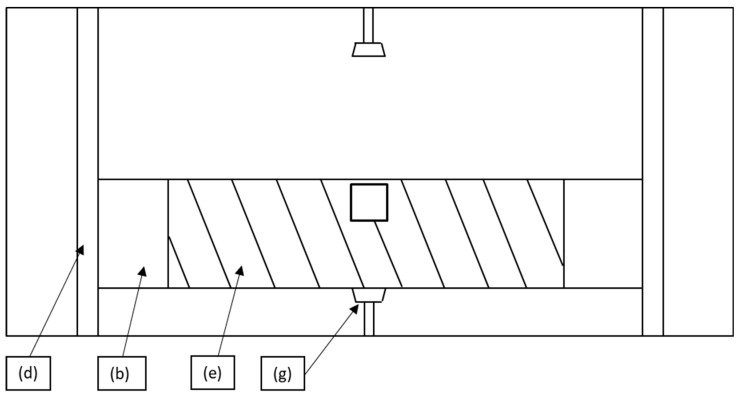
Schematic representation of the lab-scale experimental set-up seen from the top: (**b**) cavity, (**d**) guiding rods, (**e**) UD tape, (**g**) stopping pins.

Our lab-scale experimental set-up was designed to closely resemble the industrial welding process, as shown in Figure 2, where an end-of-arm tool is equipped with multiple 4 × 4 mm^2^ spot welders, which are in contact with the tapes at the beginning of the welding sequence. Schematics of the experimental set-up are shown in Figure 3 and Figure 4. The welding unit was an FLE100562 ceramic heating element (Figure 3a), the 4 × 4 mm^2^ head of which can be heated on one side to a maximum temperature of 600 °C. The custom-built energy supply was controlled by a Type K thermocouple positioned inside the welding head, with a maximum error of 3.75 °C (at 500 °C welding temperature with a 0.75% error) and a minimum error of 2.2 °C. The ceramic element itself was heated by resistance heating with the help of a wire placed inside of the welding head. While the welding time could be adjusted, the pressure applied to the welded spot resulted from the downward movement of the welding unit.

To ensure reproducibility of the experiments in terms of the geometrical position of the welded spots, a piece of metal (Figure 3b and Figure 4b) with a 120 mm long and 50 mm wide cavity was placed between the welding unit and the table (Figure 3c). Owing to the guiding rods, the piece of metal could be moved vertically to allow multiple welds to be made (Figure 4d). The metal part was moved such that the weld points were always made at the same positions on the UD tape (Figure 4e). In the production cell, the UD tapes were laid on top of a porous material, and vacuum was applied from below to keep them in a fixed position. The same porous material was used under the welding unit (Figure 3f) to rule out potential differences in results between the set-up and the industrial process chain due to a difference in thermal conductivity between the stamping head, the tapes, and the table. The stopping pins (Figure 4g) ensured that the metal piece did not fall off during welding.

**Figure 5:**
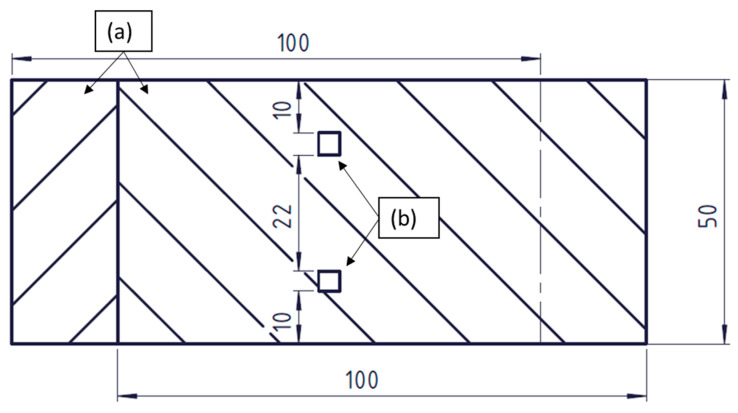
Schematic representation of the specimen: (**a**) tapes and (**b**) position of the welds.

The 4 × 4 mm^2^ welding head was used to make two spot welds on top of a stack of two UD tapes. The two welds were 22 mm apart and 10 mm away from the edges of the tapes (Figure 5).

### 3.3. Experimental Design

An overview of the experimental design is given in Table 2. The first set of experiments, the *screening test*, was based on a full factorial design using five temperatures and four welding times. Weld quality was assessed in offline tests by analyzing the size of the welded area, the maximum force during a tensile test, and the tensile stress.

The second set of experiments, the *optimization test*, also included a full factorial DoE, but with adjusted process settings. The modifications to the parameters consisted of finer discretization of (i) the welding time by 0.5 s and (ii) the temperature by 25 °C. In addition, between the welding temperatures of 325 °C and 350 °C, more samples were produced at 5 °C increments. To ensure process stability, the welding unit was cleaned after each weld by increasing the temperature of the unit to 600 °C for at least 30 s. This approach was based on conclusions drawn from the screening test, where material that accumulated on the welding unit hindered the heat transfer between unit and material. The welded area was used as a metric to evaluate variations in the process. For both the *screening* and *optimization tests*, five samples were produced for each setting.

The final set of experiments, the *continuous test*, was conducted to optimize the welding unit for a “short-wave” process. In the process cell, the welding units are usually processed in a “long-wave” setting, where the temperature is around the processing temperature of the material and the welding time is between 1 and 3 s. In this test, the process window for the welding temperature was based on the optimized setting from the *optimization test* and on the temperature at which the matrix material burns off from the surface of the spot welder according to prior experience. The welding time was fixed to 0.25 s, and the following three parameters were changed: (i) the temperature, (ii) the time between two welding steps, and (iii) the number of UD tapes in the stack underneath the welded area. The welding time was set to the lowest presumed setting at which the real process was presumed to be stable. The time delay between two welds was of relevance to understanding whether welding in quick succession caused any instabilities. Further, we wanted to know whether welding with additional tapes beneath the spot weld would cause heat dissipation toward the tapes and thus affect the energy requirement. In these experiments, 20 spot welds were made “continuously” at the time intervals specified in the DoE and without cleaning. The aim was to see whether the size of the welded area remained the same after 20 welds. For the DoE, a face-centered central composite design (CCD) was used. Two metrics were used to analyze the results: (i) the size of the welded area on the first UD tape to assess process stability and (ii) the molecular weight distribution of the polymetric matrix measured by gel permeation chromatography (GPC) to analyze degradation of the welded area.

Table 2, Table 3 and Table 4 summarize the tests and settings used. To assess the statistical significance of our results, we applied the ANOVA statistical method (with a 95% statistical confidence). In addition to the statistical analysis, main influence graphs and the stability of the process were analyzed.

### 3.4. Mechanical Test Set-Up

**Figure 6:**
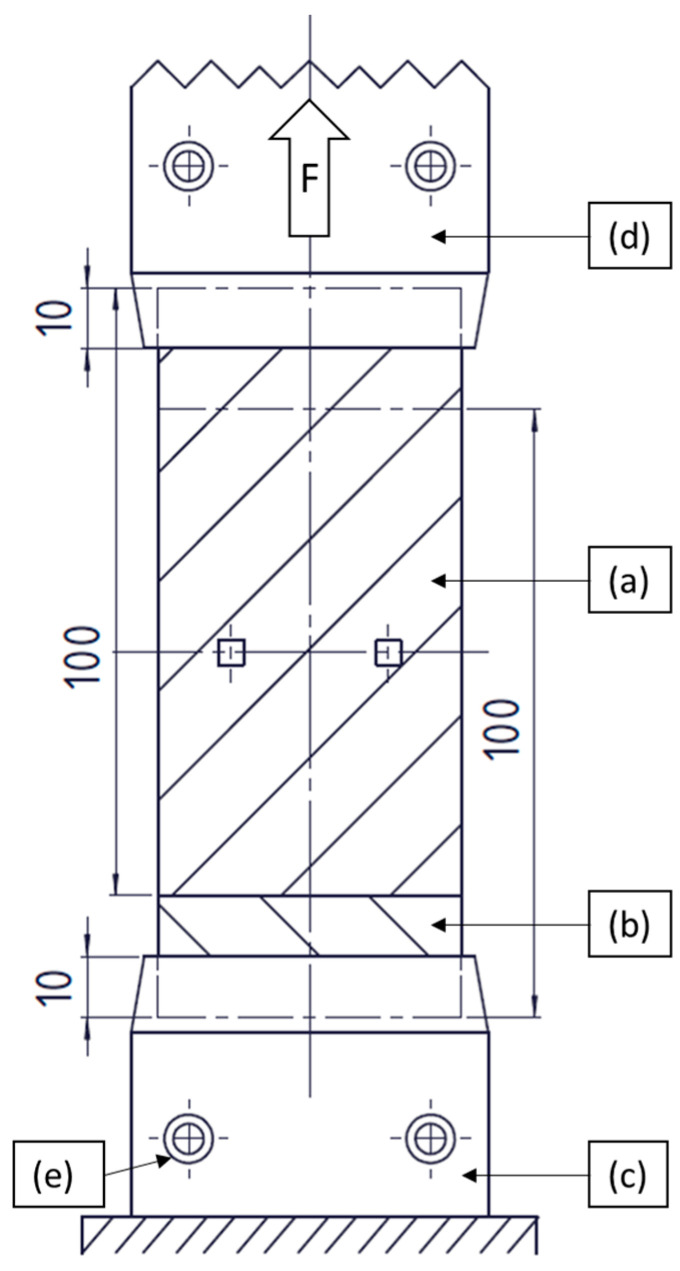
Schematic representation of the mechanical test set-up: (**a**,**b**) tapes, (**c**) fixed clamping unit, (**d**) moving clamping unit, (**e**) tightening screws.

Tensile tests were conducted with the joined UD tapes to investigate the mechanical performance of the weld. Due to the size of the welded area produced by the welding unit and the width of the UD tapes, it was deemed that a lap joint spanning the width of the UD tapes would be unreproducible and difficult to achieve, therefore, no standards were followed for the conduction of the tensile tests. An MTS 852 Test Damper System with a 10 kN tensile head was used to ensure precise results (±1 N). Figure 6 shows the schematic of the mechanical test set-up. The spot-welded tape-stack (Figure 6a,b) was placed between two mechanical clamping units (Figure 6c,d) in which each UD tape was clamped at a distance of 10 mm from each end. The tapes were secured in the clamping units with tightening screws (Figure 6e). The bottom clamping unit (Figure 6c) was fixed, while the top clamping unit (Figure 6d) applied the force to the spot-welds by moving upwards at a constant speed. Based on preliminary tests, a test speed of 1 mm/s was chosen. Particular care was taken to ensure that the welded spots were on the same horizontal plane so that the force applied to them would be perpendicular. To calculate the tensile stress at break, the maximum force was recorded. This value correlates with the moment when the welded spot fails under the load. The area of the welded spot was measured by manually selecting the corresponding area with a Keyence VHX-7000 microscope (Keyence International, Mechelen, Belgium).

### 3.5. Degradation Measurements

Degradation of UD tapes after welding was assessed by gel permeation chromatography. We analyzed the tape samples welded at 400 °C, 450 °C, and 500 °C for 0.25 s in the continuous test in terms of molecular weight of the matrix at the welded spot. Additionally, an unwelded tape was analyzed to obtain a baseline measurement for the molecular weight. For this test, no standardized norm was followed. Six spot welds from three specimen of one temperature setting were first cut out of the UD tape and were mixed into one container. Then, as a solvent, CH_2_CL_2_ was used at a rate of 26.6 g/10 mL.

## 4. Results and Discussion

### 4.1. Screening Test

**Figure 7:**
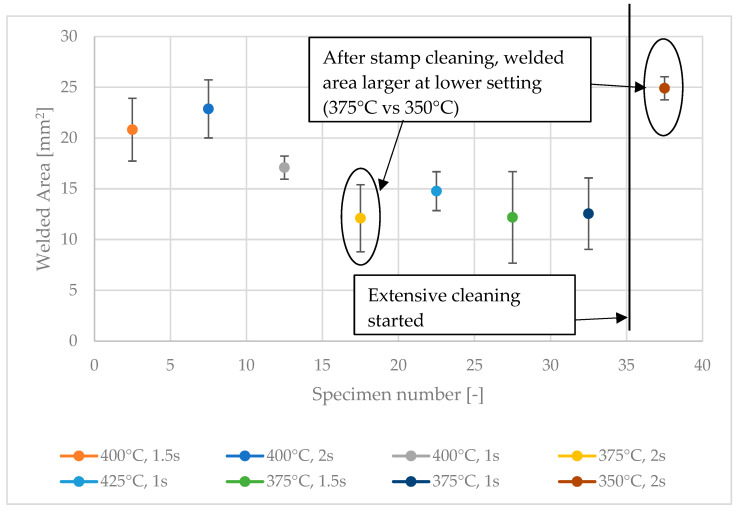
The total welded area of the first 40 samples made during the screening test at 350, 375, and 400 °C for 1, 1.5, and 2 s welding time. The vertical line indicates the time point at which the stamp welder was extensively cleaned.

Selected results of the full factorial screening tests are shown in Figure 7, which illustrates the welded area measured of the first 40 samples. For each sample, two spot welds were made, the areas of which were then added to yield the total welded area. The nominal area of the welds should be 32 mm^2^ in total according to the area of the stamp. The welded area decreased with increasing sample number: the first sample had a total welded area of 24.54 mm^2^, while that of the sixteenth sample amounted to only 7.54 mm^2^ due to contamination of the weld stamp. 

**Figure 8:**
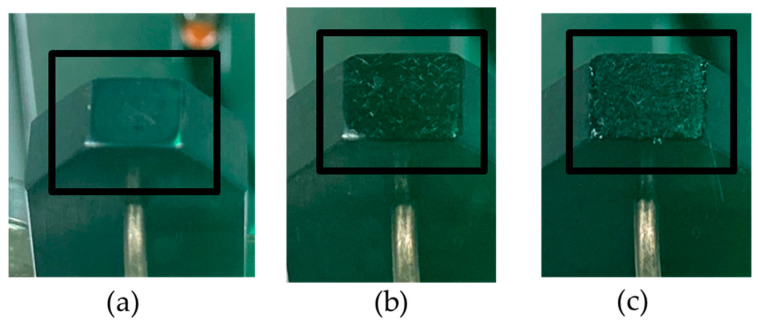
Residual matrix stuck to the welding unit after (**a**) cleaning, (**b**) after the third weld, and (**c**) after the fiftieth weld.

To investigate the contamination behavior of the stamp, several welds were made with the same process parameters. The welding unit was first cleaned by setting the temperature to 600 °C for several minutes. In Figure 8, the first photograph shows the welding head without matrix residue. A total of 50 welds, each lasting 1.5 s, were then performed at 400 °C. As soon as after the second weld, a thin layer of matrix stuck to the welding head, and this layer became thicker and more visible as more welds were performed, as can be seen in the second and third photographs in Figure 8a–c, respectively.

Contamination of the weld stamp is critical to the weld quality. As can be seen in Figure 7, after cleaning the welding head, the welded area was nearly twice the size at 350 °C than at 375 °C for the same welding time. We therefore conclude that before the cleaning procedure the welding process was inhibited by contamination of the welding head; more precisely, the matrix material sticking to the head reduced the heat transfer between welding unit and tapes.

**Figure 9:**
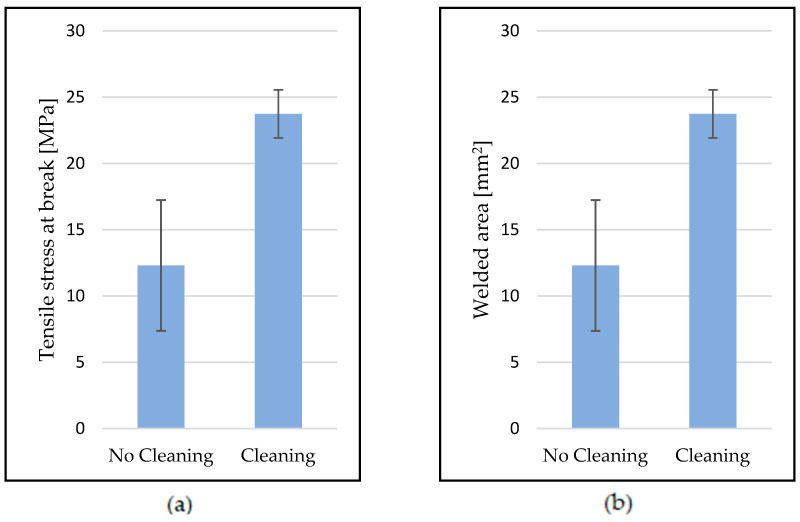
The influence of cleaning the welding head on (**a**) tensile stress at break and (**b**) welded area for samples welded at 375 °C for 1, 1.5, and 2 s (15 samples for each condition).

To further investigate the effects of contamination, the welding procedure was repeated, producing samples at 375 °C and 2 s welding time with the welding unit being cleaned before each welding cycle. Figure 9a shows the influence of stamp cleaning on tensile stress at break. When no cleaning was performed, lower tensile stress at break was recorded. In addition, the standard deviation of the results was higher than for samples that were welded after cleaning. ANOVA statistically confirmed (*p*-value of 9.64 × 10^−7^) that this effect is significant (see Table A1 in Appendix A). The welded area (Figure 9b) also indicates that a contaminated stamp reduces the heat transfer between the welding unit and the UD tape. Cleaning the stamp therefore makes the welding process more stable and results in higher-quality welds.

**Figure 10:**
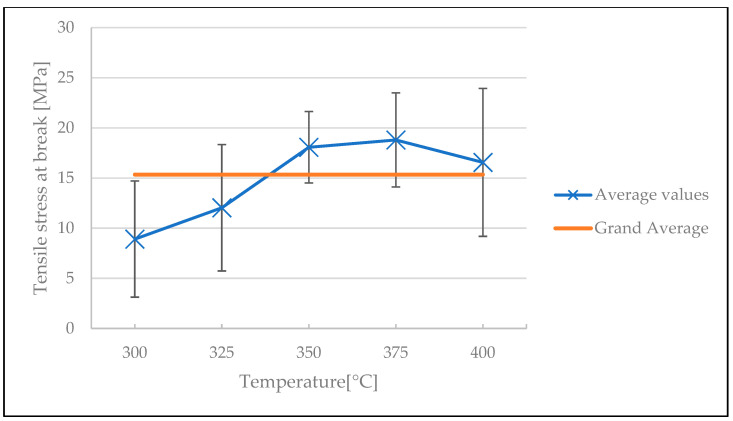
The influence of welding temperature on the tensile stress at break in the screening test.

**Figure 11:**
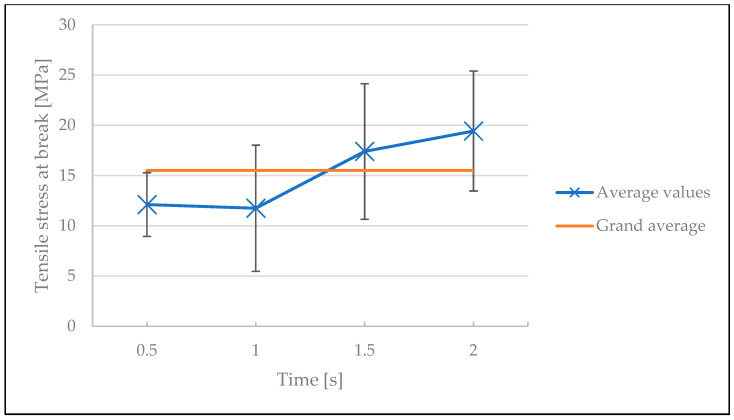
The influence of welding time on the tensile stress at break in the screening test.

The influences of welding temperature and welding time on the mechanical performance of the welded spot are illustrated in Figure 10 and Figure 11, respectively (see Table A2 and Table A3 in Appendix A for ANOVA). The samples produced prior to the cleaning procedure were remanufactured, but the stamp was cleaned after every five samples made. The standard deviation of the results was high, almost reaching the grand average value. The average tensile stress at break increased with increasing welding temperature, and at about 350 °C it started to plateau at around 19 MPa. This indicates the behavior explained by the DoB model: the mechanical performance (e.g., the inter-laminar shear strength) of a consolidated thermoplastic composite will reach a plateau, and it is impossible to further improve part quality in terms of mechanical strength [40]. Increasing the welding time also showed a slight improvement in the tensile stress at break values. Note that samples made at 300 °C and 325 °C welding temperature for 0.5 s welding time did not exhibit a stable weld and were thus not included in the analysis.

For the next experiment—the *continuous test*—the sample production and the values chosen for the process parameters were adjusted. While cleaning the spot-welder after every five samples produced improved the quality of the welds, it was still assumed that the robustness of the experiment can be further improved by cleaning the stamp after every weld. Additionally, based on the results of the screening test, we concluded that samples made at 300 °C were not stable, therefore, we substituted this setting with a higher temperature to further investigate the plateau behavior seen in Figure 10.

### 4.2. Optimization Test

**Figure 12:**
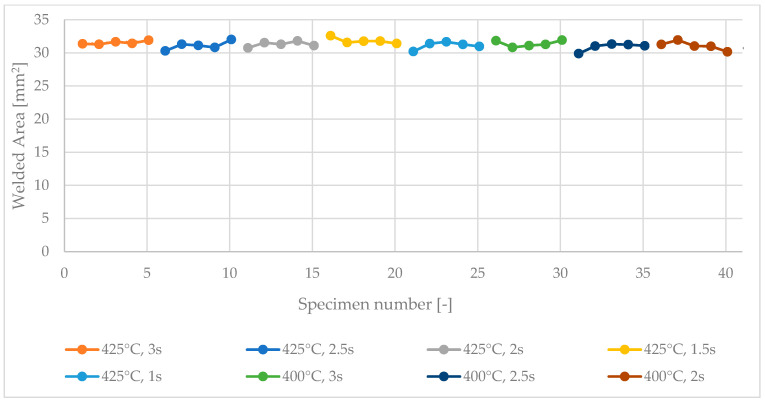
Sizes of the areas welded successively at 400 °C and 425 °C for 1, 1.5, 2, 2.5, and 3 s.

**Figure 13:**
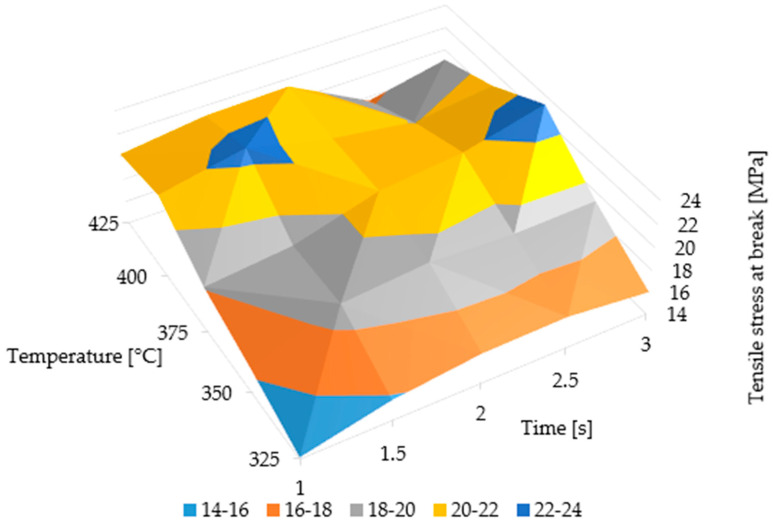
Surface plot of stress at break values of the continuous test.

Figure 12 shows the sizes of the welded areas of the first 40 samples in the optimization test. Moreover, Figure 13 illustrates a surface plot of the stress at break values obtained from the continuous test. In these experiments, the welding head was cleaned before each welding step by increasing the temperature of the welding unit to 600 °C for 30 s, which led to welded areas of roughly constant size even after multiple cycles.

**Figure 14:**
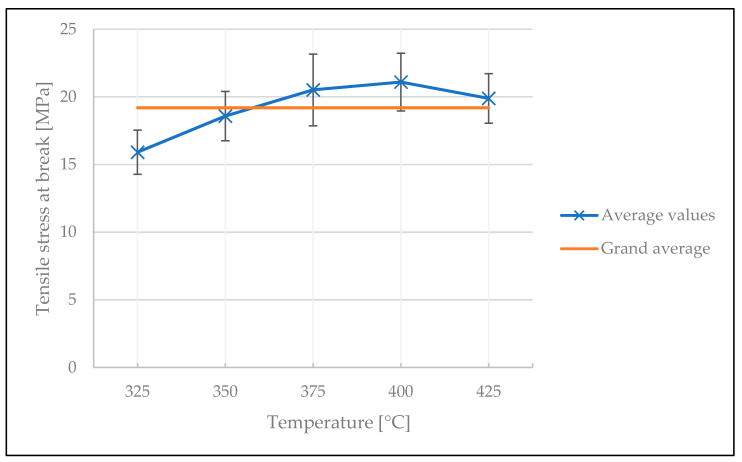
The influence of temperature on the tensile stress at break in the optimization test.

The influence of temperature on the tensile stress at break is illustrated in Figure 14. As in the screening study, the curve starts to plateau at around 350 °C, confirming the predictions of the DoB model [40]. A decrease in welding strength is evident at 425 °C welding temperature and at higher welding times. We assume that this result is caused by the degradation of the matrix material; however, within the scope of this study this was not further investigated. The welding process having been stabilized, the standard deviation of the results is also smaller than in the screening test. ANOVA showed that the influence of temperature was significant, with a *p*-value of 8.6 × 10^−15^ (see Table A4 in Appendix A). This can be explained by the rheological nature of the polymers: by increasing the temperature, the viscosity of the matrix material decreases. This in turn results in an increase in the mobility of the macromolecules which promotes the entanglement between the molecular chains.

**Figure 15:**
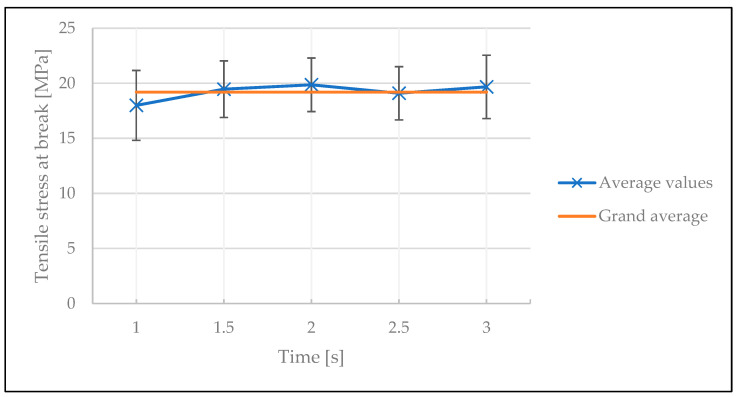
The influence of welding time on the tensile stress at break in the optimization test.

The welding time, in contrast, did not affect the quality of the weld, as the average values and their standard deviations all fell in the vicinity of the grand average of the results. ANOVA also indicated with a *p*-value of 0.12 that the effect of time was statistically insignificant (see Figure 15 and Table A5 in Appendix A). The difference of the influence of welding time between the screening and optimization tests can be explained by the different processing windows used in both experiments. In both cases, increasing the welding time at lower welding temperatures slightly increased the tensile stresses. In addition, the optimization test shows that at lower temperatures (325 °C and 350 °C), increasing the welding time slightly improves the tensile stress at break. However, these observations are within error, therefore, welding time cannot be regarded as a main influencing factor.

**Figure 16:**
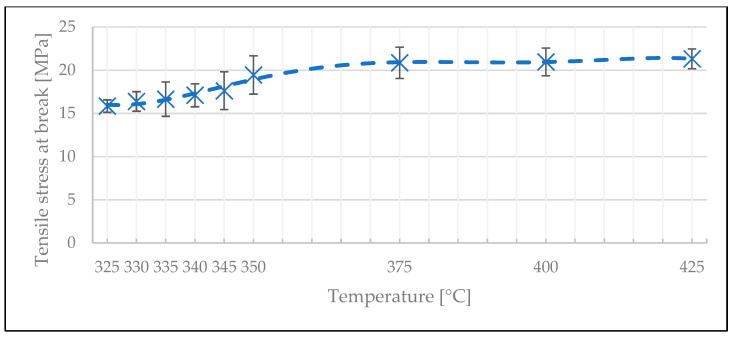
The influence of temperature on the stress at break values for 2 s welding time in the optimization test.

To further investigate the plateau behavior indicated by the degree of bonding model, the DoE was refined in the temperature range of 325 °C and 350 °C, with the welding time set to 2 s. The average stress at break values slowly increased with increasing welding temperature up to 350 °C (Figure 16). Above this temperature, the strength of the bond did not seem to increase further. We therefore conclude that the maximum weld strength that can be achieved by using the welding unit is between 20 and 23 MPa, where the degree of bonding should equal 1 [40].

The results of the optimization study can be summarized as follows: First, the welding procedure should be as fast as possible. A fast process is also economically preferable to a slow one. Second, the maximum strength achievable when using PC/CF in this welding set up is around 20 MPa. Subsequent observation of the tape laying cell during production shows that the tape-stack remains stable during transportation in the processing cell. Finally, to save energy, the temperature of the welding unit should be minimized. For these reasons, a welding temperature of 400 °C and a welding time of 1 s were defined as the optimal process settings for producing a weld of sufficient quality in the shortest time possible in terms of a long-wave process. Table A6 in Appendix A summarizes the results of the optimization test.

From a production perspective however, it can be also argued that parts produced at the lowest settings (325 °C welding temperature with a welding time of 1 s) are already optimal. Even though the mechanical strength of 15 MPa is limited, employing additional spot-welders in the highly modular end-of-arm-tool (as shown in Figure 2) and reducing the dynamic forces in the production cell by lowering the speed of the industrial robots reduces the chance of the tape-stack falling apart during transportation. To achieve this strength with a low standard deviation in a continuous process, cleaning of the spot-welders is necessary after each weld step, which is economically not feasible in an industrial environment. Therefore, to achieve the continuous cleaning procedure without intervention during a continuous production a short-wave approach was chosen for the welding procedure as a further optimization step in the *continuous test*. In this approach, the temperature was set to a higher value while the welding time was kept low to avoid excess degradation of the UD-tape. In this way, between two welding procedures in the thermoplastic composite production cell, the matrix can freely burn-off from the spot-welders thereby achieving cleaning without outside intervention.

### 4.3. Continous Test

**Figure 17:**
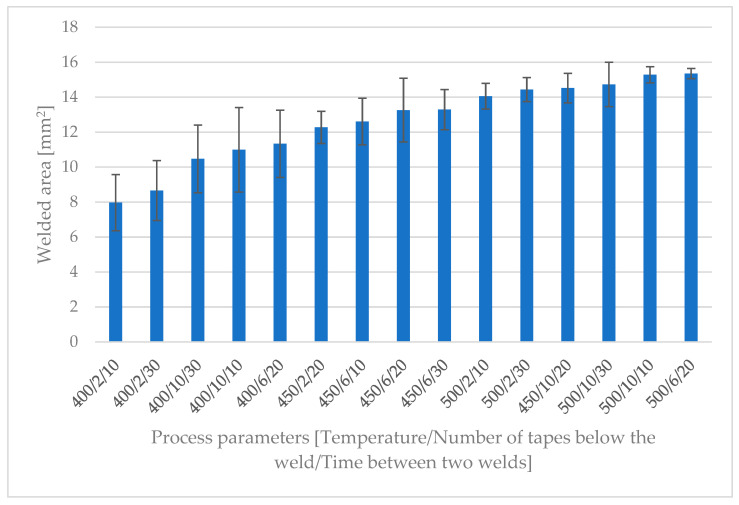
Average sizes of welded areas of 20 welds for various process settings and 0.25 s welding time in the continuous test. The bar chart is ordered by welded area in ascending order.

Figure 17 shows the average continuously welded areas obtained for various temperatures, numbers of tapes below the weld and periods between two welds when the welding time was set to 0.25 s. The graph shows only the welded area of a single spot weld, as the specimens concerned did not undergo tensile testing. As found in the optimization test, a stable and sufficiently large welded area ensures that a tensile strength value of at least 15 MPa with a low standard deviation can be achieved, which is enough to withstand the dynamic forces affecting the tape stack during production if the stamp welder is cleaned properly. The results show that the main influencing factor was the temperature. On average, at 400 °C one welded area was only between 8 and 11 mm^2^, while at 500 °C it ranged between 14 and 15 mm^2^.

**Figure 18:**
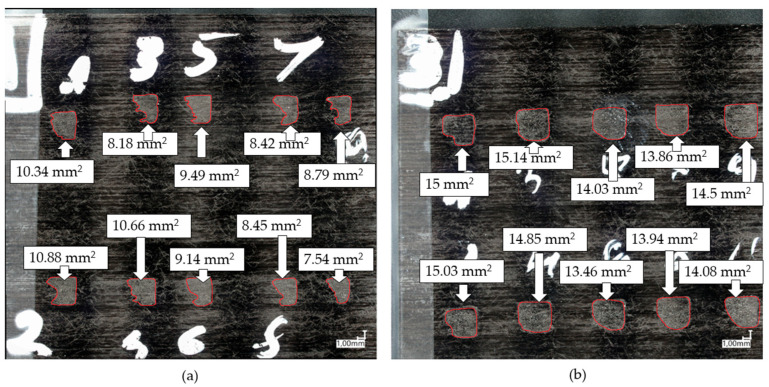
Annotated photographs of the welds made with two tapes below the weld and 10 s between two welds at (**a**) 400 °C and (**b**) 500 °C.

Figure 18 shows ten consecutive welds made at welding temperatures of 400 °C (Figure 18a) and 500 °C (Figure 18b). The first few welds were acceptable in terms of size of the welded area, but without cleaning it decreased with increasing number of welds. At 500 °C such a change in area was not apparent.

**Figure 19:**
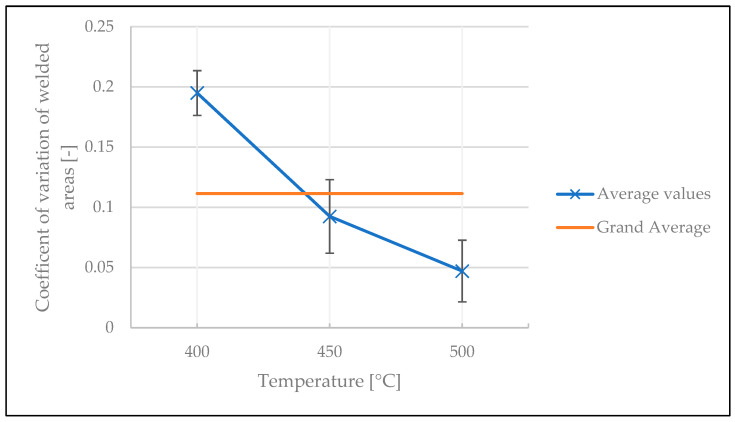
Influence of temperature on the coefficient of variation of welded areas in the continuous tests.

**Figure 20:**
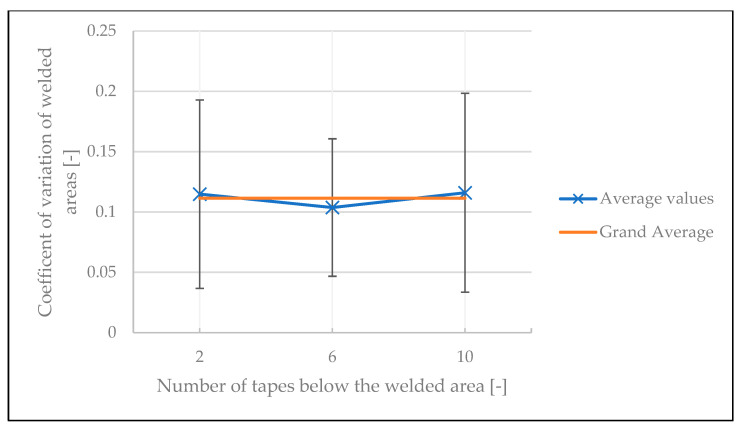
Influence of number of tapes underneath the welding on the coefficient of variation of welded areas in the continuous tests.

**Figure 21:**
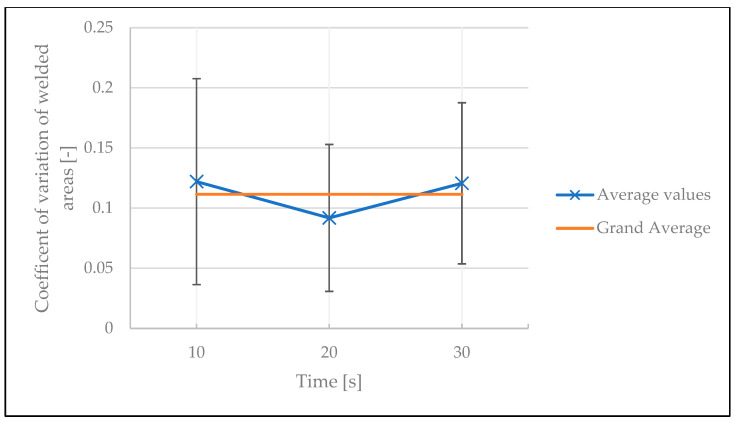
Influence of time between two welds on the coefficient of variation of welded areas in the continuous tests.

To investigate the stability of the welds, the coefficient of variation of the results was analyzed. The influences of temperature, number of tapes under the spot weld, and time between two welds on the coefficient of variation of the welded areas are illustrated in Figure 19, Figure 20 and Figure 21, respectively. The variation in welded area was greater at lower temperature than at higher temperature. This is a statistically significant result, as confirmed by ANOVA with a *p*-value of 2.82 × 10^−6^ (see Table A7 in Appendix A). For the other two factors, however, no statistically significant impact was identified (the *p*-value was 0.95 for the number of tapes below the welded area, and 0.76 for the time between two welds, see Table A8 and Table A9 in Appendix A).

**Figure 22:**
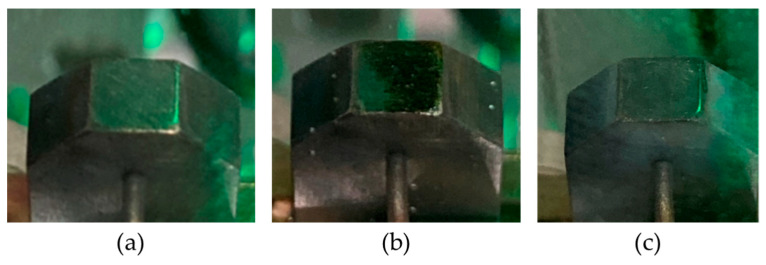
The welding head when it has been (**a**) cleaned and (**b**) after 20 welds at 450 °C and (**c**) at 500 °C for 0.25 s welding time.

To further investigate the cause of this result, three photographs were taken of the welding unit. The photographs in Figure 22a–c show, respectively, a properly cleaned stamping head, the welding unit after 20 welds at 450 °C, and the welding unit after 20 welds at 500 °C. It can be seen that at 500 °C the stamp “self-cleaned”, as it burnt off the residual matrix between two spot welds. It seems that even 450 °C was not hot enough to achieve this within the time frame of the experiment.

**Figure 23:**
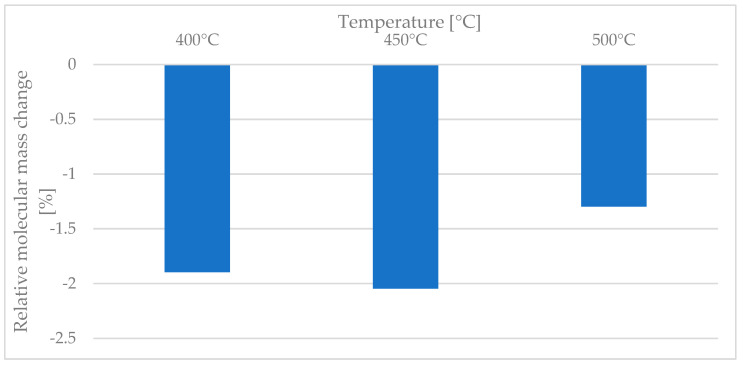
The relative change in molecular mass in relation to various welding temperatures for a welding time of 0.25 s.

One concern when employing a relatively high temperature is that it may cause the matrix material to degrade. Figure 23 shows the change in molecular mass as a function of the welding temperature. It can be seen that relative to the reference material there was a maximum decrease of only 2.04%. This change in molecular mass was independent of the temperature used. Furthermore, the size of the degraded area relative to the area of a tape-stack is at least an order of magnitude lower. We thus conclude that the degradation during a welding time of 0.25 s is negligible.

These results show that using a welding temperature of 500 °C has three advantages: First, the welded area remains constant over several welds. Although no tensile tests were performed with welds made at 500 °C, on the basis of the optimization test we consider it safe to assume that the tensile strength of such a weld—if a fully welded area was achieved—would be at least 15 MPa. Second, using this high temperature allows the welding time to be reduced to 0.25 s. The obvious benefit is the time saved, but—as previously mentioned—degradation could be a serious concern. However, we have shown that a short welding time prevents degradation of the matrix material. We assume that the low heat conductance of PC is the main reason that no degradation was observed. Lastly, keeping the welding unit at 500 °C means that any residual matrix sticking to the metal surface burns off, and thus the welding head cleans itself.

## 5. Conclusions and Outlook

This work focused on optimizing the welding procedure of PC/CF tapes with a spot-welding unit to obtain a quality weld with reduced cycle time for the welding production process in the first stage of the thermoplastic production cycle. Three separate studies were conducted to find the optimal process parameters.

In the screening test, it was shown that failing to clean the stamp welder made the process unstable, as the heat transfer between head and tape became restricted. Both here and in the optimization test, we found that the maximum mechanical performance of a weld attainable by the stamp welding unit was achieved at welding temperatures above 350 °C and welding times between 1 and 2 s. The maximum achieved tensile stress at break was around 23 MPa. We additionally showed that the welding temperature had the strongest impact on the tensile stress at break. This is due to the increased molecular mobility of the macromolecules, which in turn results in higher level of entanglement of molecular chains.

The stability of the process is as important as the mechanical performance achieved by spot-welding. Based on the welded area, we concluded that steady state processing conditions can be attained by continuous cleaning of the stamp welder. The continuous test demonstrated that the process can be further improved and stabilized by employing a welding temperature of 500 °C for 0.25 s welding time. In this case, the process was much faster, the welding unit self-cleaned, and degradation was avoided due to the short welding time.

The results presented in this work were obtained for PC/CF tapes. Future studies might address the impact of the matrix material on the spot-welding process. Using the “short-wave” approach might be detrimental for other matrix materials with distinct degradation mechanisms. Furthermore, the number of spot welds needed to withstand the dynamic forces in the production cell was not explored. By defining a minimum required number of welds, the process can be further optimized by limiting the number of spot welders employed which could result in lower energy usage and costs.

## Figures and Tables

**Table 1:** Properties of the matrix and fiber materials in the UD tape.

Material	Property	Value
Matrix	Melt mass-flow rate [g/10 min]	37
	Density [kg/m^3^]	1190
	Glass transition temperature [°]	145
	Tensile modulus [MPa]	2400
	Yield stress [MPa, at 50 mm/s]	65
Fiber	Density [g/cm^3^]	1.82
	Denier [den]	14,400
	Tensile modulus [GPa]	36

**Table 2:** Summary of the screening test, listing the process parameters varied in each test, the values chosen, and the metrics used for assessment.

Process Parameters	Values	Metrics
Temperature [°C]	300 325 350 375 400	Welded area Maximum force Tensile stress
Welding time [s]	0.5 1 1.5 2	

**Table 3:** Summary of the optimization test, listing the process parameters varied in each test, the values chosen, and the metrics used for assessment.

Process Parameters	Values	Metrics
Temperature [°C]	325 350 375 400 425 At 2 s weld time: 330 335 340 345	Welded area Maximum force Tensile stress
Welding time [s]	1 1.5 2 2.5 3	

**Table 4:** Summary of the continuous test, listing the process parameters varied in each test, the values chosen, and the metrics used for assessment.

Process Parameters	Values	Metrics
Temperature [°C]	400 450 500	Welded area Gel Permeation Chromatography
Time between two welds [s]	10 20 30	
Number of tapes below the weld [-]	2 6 10	

## Data Availability

The data presented in this study are available on request from the corresponding author.

## Abbreviations

The following abbreviations are used in this manuscript:

ANOVA	Analysis of variance
CCD	Central composite design
DoB	Degree of bonding
DoE	Design of experiments
FTIR	Fourier transform infrared
GPC	Gel permeation chromatography
PC	Polycarbonate
PC/CF	Polycarbonate/carbon fiber
PEEK	Polyether ether ketone
UD	Unidirectional

## Appendix A

**Table A1:** ANOVA of the influence of cleaning the stamp welder.

ANOVA						
*Source of Variation*	*SS*	*df*	*MS*	*F*	*p-value*	*F crit*
Between Groups	519.8481	1	519.8481	41.46277	9.64 × 10^−7^	4.241699
Within Groups	313.4427	25	12.53771			
Total	833.2908	26				

**Table A2:** ANOVA of the screening test in terms of welding time.

ANOVA						
*Source of Variation*	*SS*	*df*	*MS*	*F*	*p-value*	*F crit*
Between Groups	1112.744	4	278.1861	8.701923	6.47 × 10^−6^	2.481661
Within Groups	2653.373	83	31.96835			
Total	3766.117	87				

**Table A3:** ANOVA of the screening test in terms of welding temperature.

ANOVA						
*Source of Variation*	*SS*	*df*	*MS*	*F*	*p-value*	*F crit*
Between Groups	971.9521	3	323.984	9.739818	1.39 × 10^−5^	2.713227
Within Groups	2794.165	84	33.26387			
Total	3766.117	87				

**Table A4:** ANOVA of the optimization test in terms of welding temperature.

ANOVA						
*Source of Variation*	*SS*	*df*	*MS*	*F*	*p-value*	*F crit*
Between Groups	412.0432	4	103.0108	24.6047	8.6 × 10^−15^	2.449202
Within Groups	489.836	117	4.186632			
Total	901.8792	121				

**Table A5:** ANOVA of the optimization test in terms of welding time.

ANOVA						
*Source of Variation*	*SS*	*df*	*MS*	*F*	*p-value*	*F crit*
Between Groups	53.48749	4	13.37187	1.825407	0.12853	2.449202
Within Groups	857.074	117	7.325419			
Total	910.5615	121				

**Table A6:** Summary of stress at break values of the optimization test. The selected process setting is indicated by a black border.

Temperature [°C]	Time [s]	Average Stress at Break [MPa]	Standard Deviation of Stress at Break [MPa]
325	1	14.17	1.06
325	1.5	15.85	0.71
325	2	16.71	2.2
325	2.5	16.84	1.77
325	3	16.03	0.89
350	1	16.28	1.84
350	1.5	18.83	1.28
350	2	19.46	2.22
350	2.5	19.1	0.97
350	3	19.25	0.81
375	1	18.07	1.66
375	1.5	18.83	0.65
375	2	20.86	1.81
375	2.5	21.57	2.7
375	3	23.26	3
400	1	21.21	3.08
400	1.5	22.66	1.38
400	2	20.96	1.61
400	2.5	19.92	1.67
400	3	20.74	2.37
425	1	20.24	1.29
425	1.5	21.14	1.26
425	2	21.32	1.15
425	2.5	17.65	1.06
425	3	19.09	1.53

**Table A7:** ANOVA of the influence of temperature on the coefficient of variation of welded areas.

ANOVA						
*Source of Variation*	*SS*	*df*	*MS*	*F*	*p-value*	*F crit*
Between Groups	0.057349	2	0.028674	44.47129	2.82 × 10^−6^	3.885294
Within Groups	0.007737	12	0.000645			
Total	0.065086	14				

**Table A8:** ANOVA for the influence of number of tapes underneath the welded area.

ANOVA						
*Source of Variation*	*SS*	*df*	*MS*	*F*	*p-value*	*F crit*
Between Groups	0.00046	2	0.00023	0.042682	0.958361	3.885294
Within Groups	0.064626	12	0.005386			
Total	0.065086	14				

**Table A9:** ANOVA for the time between two welds on the coefficient of variation of welded areas.

ANOVA						
*Source of Variation*	*SS*	*df*	*MS*	*F*	*p-value*	*F crit*
Between Groups	0.002902	2	0.001451	0.280015	0.760578	3.885294
Within Groups	0.062184	12	0.005182			
Total	0.065086	14				
